# The modulation effect of cognition on the interpretation bias of mentalization in late-life depression (LLD): A study of eye gaze interpretation – a potential screening tool for high-risk group of LLD

**DOI:** 10.1192/j.eurpsy.2024.1318

**Published:** 2024-08-27

**Authors:** P. W. Cheng

**Affiliations:** Psychiatry, the university of Hong Kong, Hong Kong, Hong Kong

## Abstract

**Introduction:**

Impairment in mentalization, interpreting and perceiving social relevant information has been found to play a part in the development and maintenance of depression. Major depressive disorders showed significant impairment in social cognition and such impairment appears to be positively associated with the severity of depression. Self-referential gaze perception, as a measurement of mentalization, was predominantly measured in patients with psychosis but rarely examined in late-life depression (LLD).

**Objectives:**

To assess the effect of cognition on the interpretation bias of mentalization

**Methods:**

This will be a cross-sectional case-controlled study on Chinese older adults with major depressive disorder recruited from outpatient departments of the public mental health service in Hong Kong. The same inclusion and exclusion criteria, with the exception of the history of major depressive disorder, will be used to recruit the control group. Assessments included sociodemographics, cognitive assessments and depressive symptoms. The primary experimental task was Gaze Perception Task using E- prime Professional 2.0. The stimuli of task are photographs of six Chinese models (3 men and 3 women) facing straight to camera with 13 different gaze directions (0°, 5°, 10°, 15°, 20°, 25° and 30° to the left and to the right, respectively). Participants shall be instructed to respond with a “yes” or a “no” to the question (for self-referential gaze): ‘Do you feel as if the person in the picture is looking at you?’.

**Results:**

41 patients and 41 healthy controls have been recruited. The group comparison in SRGP revealed that there was only significant difference in the unambiguous-SRGP (U=561.000, Z=-2.62, N=82, p=0.009). Patients had higher unambiguous self-referential gaze accuracy (Mean=0.16) than controls (Mean= 0.075). With a cut-off score of 22, patients with better HK-MoCA scores had better unambiguous SRGP scores than those with lower HK-MoCA scores (p=0.024). This difference was not observed in healthy controls. HK-MoCA could predict ambiguous SRGP rate F(1,80)=14.85, p<.001, R square=15.7%. and redict unambiguous SRGP rate F(1,80)=14.85, p<.001, R square=15.7%.

**Conclusions:**

LLD subjects had a significant interpretation bias in the unambitious averted gaze (20°, 25° and 30°) interpretation compared with healthy controls. LLD subjects tend to have more self-referential perception of the clear averted gaze. This misinterpretation of the eye gaze is probably due to the interpretation bias in processing external information, which is commonly reported as mentalization impairment in depression (Weightman et al., 2014).

**Disclosure of Interest:**

None Declared

